# Risk factors for iatrogenic retinal breaks during induction of posterior vitreous detachment in macula surgery

**DOI:** 10.1007/s10792-026-04162-z

**Published:** 2026-07-12

**Authors:** Jai Ethan Paris, Carmelo Zak Macri, Surbhi Agrawal, Stewart Lake, Weng Onn Chan

**Affiliations:** 1 Discipline of Ophthalmology and Visual Sciences, The University of Adelaide, Adelaide, SA 5005 Australia; 2 Department of Ophthalmology, The Royal Adelaide Hospital, Port Road, Adelaide, SA 5000 Australia; 3 Department of Ophthalmology, Flinders Medical Centre, Adelaide, SA Australia

**Keywords:** Posterior vitreous detachment, Retinal break, Vitrectomy, Surgical technique, Retinal detachment

## Abstract

**Purpose:**

To determine risk factors for retinal breaks (RB) during the surgical induction of posterior vitreous detachment (IPVD) in macula surgery.

**Methods:**

We retrospectively reviewed electronic records for all adult patients undergoing 25-G or 27-G vitrectomy for macula pathology between November 2019 and January 2024 requiring intraoperative IPVD. We excluded eyes with pre-existing PVD, panretinal photocoagulation, trauma, age < 18 years or missing information. The outcome measure was non-sclerotomy-related iatrogenic RB. We assessed the effect of age, gauge size (25-G vs 27-G), surgical indication (epiretinal membrane [ERM] vs macular hole[MH]/vitreomacular traction syndrome[VMTS]), lens status (Phakic vs Aphakic/Pseudophakic) and surgeon (Consultant vs Fellow) on the odds of RB.

**Results:**

Among 185 eyes included in our study, 15.1% experienced a RB during IPVD. Breaks were predominantly located in the inferior retina (69%). Multivariable analysis revealed gauge size significantly impacted RB rate (27-G OR 0.13 95%CI 0.03–0.60, *p* = 0.008). Other factors including age (OR 0.98 95%CI 0.96–1.01, *p* = 0.18), lens status (Phakic OR 0.36 95%CI 0.13–1.01, *p* = 0.053), surgeon grade (Fellow OR 1.8 95%CI 0.18–18.9, *p* = 0.61) and surgical indication (MH OR 0.70 95%CI 0.30–1.7, *p* = 0.42) were not significantly associated with odds of RB.

**Conclusions:**

There is a high rate of iatrogenic RB in eyes requiring IPVD undergoing macula surgery. Smaller instrument gauge size was associated with lower odds of RB during IPVD, however causal inferences cannot be drawn from these findings. Neither age, surgical indication nor lens status were associated with odds of RB.

## Introduction

Induction of posterior vitreous detachment (IPVD) is a risk factor for retinal breaks (RB) during pars plana vitrectomy (PPV) [1–5]. Reported RB rates during IPVD range from 2.8 to 32.3%, which is considerably higher compared to break rates of 0.6–3.1% observed across all vitrectomy procedures [1, 2]. Furthermore, the presence of an existing PVD has been shown to lower the rate of RB during PPV across all gauge sizes and surgical indications including macular hole (MH), epiretinal membrane (ERM) and tractional retinal detachment (RD) [1, 6]. Some evidence also suggests that IPVD may increase the rate of postoperative rhegmatogenous RD, and this is thought to be due to missed intraoperative breaks [2].

Despite well-established literature for IPVD as a risk factor for RB during PPV, few studies explore factors that may influence this risk [7]. There is conflicting evidence regarding the impact of surgical indication on the risk of RB. Some studies suggest ERM surgery confers a higher RB rate [1, 2]. There has been significant interest regarding differences in the effect of cannula size on RB rates in eyes undergoing vitrectomy. Most studies show higher break rates in larger gauge, higher vacuum setting surgeries, however, some show no impact [4, 8–10]. In recent years there has been growing evidence supporting the safety and efficacy of 27-G vitrectomy, with multiple studies revealing effective use across several vitreoretinal conditions, offering reduced wound sealing-related complications and faster healing, greater precision in membrane dissection, improved final visual outcomes, with only slight differences in operation times [11–13]. Despite this, few studies specifically explore how 27-G surgeries compare with the more commonly used 25-G systems exclusively in eyes requiring IPVD.

Prior studies often include all vitrectomies, the majority of which do not require IPVD, making the evidence within IPVD subgroups scarce. The evidence that does exist regarding the impact of surgical indication and gauge size is limited by small-scale studies. Few other factors that might affect RB rate in IPVD have been explored in the literature. We therefore sought to identify risk factors for retinal breaks from a retrospective cohort of vitrectomies undergoing macula surgery requiring IPVD, and to evaluate the nature and characteristics of break location in these eyes.

## Methods

We reviewed the electronic medical records of all adult patients who underwent vitrectomy surgery between November 2019 and January 2024 at the Royal Adelaide Hospital and The Queen Elizabeth Hospital, Adelaide, South Australia. Ethics committee approval was obtained from the Central Adelaide Local Health Network Human Research Ethics Committee (reference number 19813) and the study adhered to the tenets of the Declaration of Helsinki.

We included all eyes which underwent pars plana vitrectomy for macula hole (MH) vitreomacular traction syndrome (VMTS), and epiretinal membrane (ERM) surgery and required intraoperative induction of posterior vitreous membrane detachment, with follow up of at least 1 month. We excluded eyes with pre-existing complete or partial PVD, those without documentation of induction techniques or intraoperative findings, eyes with previous penetrating or blunt trauma, paediatric cases (age < 18 years), and eyes with previous panretinal photocoagulation.

All patients underwent thorough preoperative assessment including an examination for best corrected visual acuity (BCVA), slit lamp examination and dilated fundus examination.

Three port PPV was performed using a 25-, or 27-G vitrectomy system (Constellation Vision System, Alcon). Sclerotomies were placed 4 mm posterior to the limbus in phakic eyes and 3.5 mm in pseudophakic eyes. An infusion cannula was positioned in the inferotemporal quadrant, and supertemporal and superonasal sclerotomies were made near the 10- and 2-o’clock meridians. After a core vitrectomy, absence of PVD was confirmed intraoperatively with intravitreal triamcinolone acetonide (IVTA). Induction was then performed by engaging the pre-papillary hyaloid into the cutter mouth in suction-only mode at a vacuum level of 650 mm Hg. The PVD was then extended using the vitrectomy probe in a cutting mode off followed by peripheral vitreous removal in cutting on mode. Peripheral vitrectomy was performed using a fixed cut rate of 5000 cuts per minute (cpm). Foot-pedal controlled proportional vacuum between 300 and 500 mmHg was used during core vitrectomy and between 20 and 300 mmHg during PVD extension and peripheral vitreous removal. Surgical procedures varied after vitreous removal, depending on the pathology. To confirm that RBs occurred during IPVD, a thorough intraoperative 360-degree peripheral search was performed immediately before and after induction of PVD.

The primary outcome was the occurrence of one or more non-sclerotomy-related RB. RBs were diagnosed by a careful 360-degree peripheral search of the retina using the Resight viewing system coupled with a Lumera 700 microscope. Definite RB had characteristics including an elevated retinal flap with a discrete edge. Breaks without a definite flap, edge or retinal defect observed during scleral indentation were excluded from the present study. Sclerotomy-related retinal breaks were defined as breaks occurring intraoperatively that were located near 1 of the 3 sclerotomies (within 1 h either side of the sclerotomy site) and were recorded but excluded from the main analysis. We included any breaks identified up to 1 month following surgery that fit these criteria. All iatrogenic breaks were treated with laser photocoagulation. If breaks were round or had significant underlying pigment surrounding them, they were categorised as pre-existing and excluded from the study.

Data collected included demographic, clinical, surgical and post-operative findings. Patient demographics included age and sex, ocular history, prior surgical history in subject eye, history of retinal breaks or detachment, and history of diabetes with proliferative diabetic retinopathy or tractional retinal detachment. Clinical factors included laterality, phakic status, intraocular pressure (IOP), BCVA (Snellen in metres). Surgical factors included the surgery indication, surgeon seniority/grade, instrument gauge size, retinal breaks, if encountered, including their location and whether they were sclerotomy-related or breaks elsewhere.

Continuous variables such as age were described using mean and standard deviation (SD) or median and interquartile range (IQR). Categorical variables such as gender, surgical indication, instrument gauge size, and surgeon grade, were summarized using frequencies and percentages.

Modified generalized estimating equations logistic regression (to account for inter-eye correlation) was used to explore the association of exposures with odds of RB. Exposure variables were adjusted for the precision variable age, identified a priori from literature as a previously known risk factor and was the minimally sufficient adjustment set for all exposure of interest after the construction of directed acyclic graphs to map potential variable relationships [14]. We assessed the direct effect of age, gauge size (25-G vs 27-G), surgical indication (MH/VMTS vs ERM) and phakic status (Phakic vs Pseudophakic) on odds of RB, adjusted for age. Data reduction of the surgical indication variable was performed by combining VMTS and MH, and with phakic status by combining aphakia and pseudophakia. We used a bias-corrected estimator in the models. Results were reported as odds ratios (ORs) with corresponding 95% confidence intervals (CIs).

All statistical analyses were performed using the statistical programming language R (packages geessbin v0.1.2, glmtoolbox v0.1.11) [15], and a *p*-value < 0.05 was considered statistically significant. *P* values were not adjusted for multiple testing due to the study’s exploratory nature.

## Results

We included 185 eyes of 180 patients in the study cohort (Table 1). The cohort included 100 females (56%) and 80 males (44%). The median age was 71 (Range 20–94, IQR 64–77).

**Table 1:** Clinical and surgical characteristics of included eyes

Characteristic	No RB, N = 157	RB, N = 28
*Laterality*		
Left	79 (50%)	10 (36%)
Right	78 (50%)	18 (64%)
*Prior intraocular surgery (excluding cataract)*		
No	155 (99%)	28 (100%)
Yes	2 (1.3%)	0 (0%)
*Phakic status*		
Aphakic	0 (0%)	0 (0%)
Phakic	134 (85%)	20 (71%)
Pseudophakic	23 (15%)	8 (29%)
BCVA (logMAR)	0.70 (0.50, 1.00)	0.70 (0.48, 1.25)
*Surgical indication*		
ERM	55 (35%)	12 (43%)
MH/VMTS	102 (65%)	16 (57%)
*Surgeon grade*		
Consultant	10 (6.4%)	1 (3.6%)
Fellow	147 (94%)	27 (96%)
*Gauge size*		
25G	100 (64%)	26 (93%)
27G	57 (36%)	2 (7.1%)
*Previous RB*		
No	156 (99%)	28 (100%)
Yes	1 (0.6%)	0 (0%)

*BCVA* best corrected visual acuity; *RB* retinal break.

There were 154 phakic eyes (83%) and 31 pseudophakic eyes (17%). There were 2 eyes with other previous intraocular surgery excluding phacoemulsification and intraocular lens implant, which included 1 case of trabeculectomy and 1 case of extracapsular lens extraction following traumatic cataract. The median preoperative BCVA was 0.70 (IQR 0.5–1.00) logMAR, i.e. 6/30 (IQR (6/19–6/60).

Vitrectomies were performed either by a fellow retinal surgeon in 174 (94%) of cases, or a consultant retinal surgeon in 11 (6.0%) cases. The indication for surgery was MH in 61%, ERM in 36%, VMTS in 6 (3.2%). The cannulation gauge used was 25-G in 68%, 27-G PPV in 32%.

In total there were 42 peripheral RB that occurred in 28 eyes during induction of PVD (Table 1). All breaks occurred intraoperatively, none were detected in the postoperative follow up. Of the 42 breaks, 14 were described as inferior, 7 were inferonasal, 8 were inferotemporal, 4 were superotemporal, 1 nasal, 3 were superonasal, 1 were temporal, 1 was peripheral, and 3 were not reported. There were 3 cases of sclerotomy-related RBs which occurred in three eyes (1.6%). These were temporal, superotemporal, and superonasal.

Of the eyes that experienced peripheral RB, 26 (93%) had 25-G surgery and 2 (7%) had 27-G surgery. The RB rate for 25-G and 27-G cannulation systems was 20.6% and 3.4%, respectively. Most RB occurred in phakic eyes (71%) of cases. The presence of RB by surgical indication was 13% for MH, 18% for ERM, 17% for VMTS.

Increasing age was not associated with odds of RB (OR 0.98, *p* = 0.18, Table 2). Gauge size of 27-G compared to 25-G was associated with lower odds of RB in unadjusted and adjusted analyses (Adjusted OR 0.13, *p* = 0.008). Neither lens status, surgical indication, nor surgeon grade was associated with odds of RB (Table 2).

**Table 2:** Multivariable generalised estimating equations logistic regression estimating the direct effect of hypothesised exposures on the odds of RB during IPVD

Factor	Unadjusted OR (95% CI)	*p* value	Adjusted OR (95% CI)	*p* value
Age	0.98 (0.96–1.01)	0.18		
*Gauge size*				
25G	Reference		Reference	
27G	0.14 (0.03–0.61)	0.009	0.13 (0.03–0.60)	0.008
*Surgical indication*				
ERM	Reference		Reference	
MH/VMTS	0.72 (0.31–1.7)	0.45	0.70 (0.30–1.7)	0.42
*Lens status*				
Pseudophakic	Reference		Reference	
Phakic	0.43 (0.16–1.1)	0.09	0.36 (0.13–1.01)	0.053
*Surgeon grade*				
Consultant	Reference		Reference	
Fellow	1.8 (0.18–18.3)	0.61	1.8 (0.18–18.9)	0.61

*OR* Odds ratio; *CI* confidence interval; *G* gauge; *ERM* epiretinal membrane; *MH* macular hole; *VMTS* vitreomacular traction syndrome; *IPVD* Induction of posterior vitreous detachment; Adjusted models are adjusted for age (as a linear variable).

## Discussion

IPVD is a known risk factor for RB during PPV [1, 4, 10]. The rate of IPVD-associated RB in our cohort of eyes was 15%, with 69% of these breaks occurring in the inferior retina. The frequency of RB in our eyes was in keeping with past evidence in similar patient populations, which report rates of 9.02–32.1% [1, 8, 14]. A recent meta-analysis of 32 studies all eyes undergoing vitrectomy found a RB incidence of 12.78% for 20-G surgery (771 eyes per 6033 cases) and 6.29% for 23-G/25-G surgeries (330 eyes per 5242 cases) [16]. They reported higher rates of RBs for eyes requiring IPVD which were subgrouped in 4 studies (14.4% for 20-G; 9.95% for 23-G/25-G). Our rate of RBs was likely higher than pooled evidence given 94% of surgeries were performed by fellows in training, which is reflective of routine case distribution at our centers. We found that smaller cannulation gauge size was associated with lower odds of RBs in our cohort. Other demographic, patient and procedural factors including age, lens status, surgical indication and surgeon grade were not associated with odds of RB.

There is conflicting evidence in the literature regarding the impact of cannulation size on the rate of RB during IPVD for macula surgery. Some studies have demonstrated similar findings to ours, suggesting larger gauge size may be associated with odds of RB rates in eyes requiring IPVD. Nakano et al. found that larger 20-G surgery was associated with a higher RB rate compared to 23-G, in a series of 225 eyes requiring ERM surgery and 103 requiring MH surgery [8]. Vitreous cut rate was fixed at 2000 cpm with maximum aspiration pressure up to 500 mmHg for both 20-G and 23-G when inducing PVD. In eyes requiring IPVD, the incidence of iatrogenic RB was 15.8% in the 20-G group and 3.1% in the 23-G group (*p* = 0.0234), however authors failed to discriminate between sclerotomy related breaks and breaks elsewhere. Jalil et al., found 20-G surgery was associated with higher odds of RBs in a large series of eyes requiring IPVD when compared to those undergoing 23-G surgery (30.5% vs. 12.8%) [9]. In contrast, Hikichi et al. performed a single surgeon retrospective review in a series of 183 eyes undergoing either MH or ERM surgery and found equal rates of RB between 20-G and 23-G surgery (20/122 (16%) vs. 10/61 (16%)) [17]. Sandali et al. reported comparable rates of RB across 553 eyes undergoing ERM surgery requiring IPVD using either 20-G, 23-G or 25-G cannulation, however no statistical analysis of the IPVD subgroup was performed (20-G 12.34% vs. 23-G 13.9% vs. 25-G 5.3%) [10]. Paris et al. previously showed smaller gauge surgeries (27-G) were associated with lower RB rates only in MH surgeries, possibly reflecting the greater requirements for IPVD and peripheral vitreous manipulation in MH procedures compared with ERM [18]. The findings of gauge related effects across previous studies are limited by small numbers of eyes requiring IPVD and the absence of IPVD subgroup analyses. Similarly, in our series, only two RBs occurred in eyes undergoing 27-G surgeries which limits the precision and stability of the estimated effect size and observed association, as reflected by wide confidence intervals. These findings should be interpreted cautiously and regarded as exploratory, and do not imply a causal relationship. Gauge size may influence RB rates through various mechanisms. Sclerotomy related breaks are due to incarceration of vitreous gel around sclerotomy sites, increasing retinal traction [8, 19]. Iatrogenic non-entry site peripheral breaks on the other hand, are generally considered secondary to the creation and extension of PVD intraoperatively, although their exact pathogenesis is incompletely understood [7]. Extrapolations from studies comparing 20-G with 23/25-G, suggest higher vacuum settings and larger port sizes of larger gauge instruments may increase retinal traction and the propensity for RB development [7, 20]. However, in our series of eyes vacuum settings for both 25-G and 27-G surgery was set at 650 mmHg. The slower rate of PVD induction with 27-G may contribute to a lower association with RB odds. Possibly the reduced cut diameter and lower duty cycle in 27-G surgery which improve fluid dynamics may also contribute to reduced odds of RBs. Previous studies have demonstrated lower IOP fluctuations in 27-G vitrectomy compared to 25-G, regardless of the aspiration system used [21]. The higher flow limitations of cutter gauge size, port size and duty cycle may be an advantage with 27-G surgeries, whereby there is reduced average vitreous fibre travel between cuts, limiting the traction exerted on the retina, and RB risk [22, 23]. The increase in fluid turbulence in 25-G may also affect retinal stability and increase the risk of RB [23]. 25-G instruments have higher rigidity compared to 27-G, which is considered desirable given they provide more control. In retinal peripheries increased flexibility may cause instrument bending, however given this is where most RB occur, more gentle retinal manipulation with less rigid instrumentation may reduce RB risk [24].

There has been increasing interest in the safety and efficacy of 27-G instruments compared with conventional 25-G microincision systems, with multiple studies citing improved intraoperative and postoperative wound-related complications, similar operation times, and possibly less postoperative IOP fluctuations [13, 25, 26]. Ma et al. performed a systematic review of 11 studies comparing the outcomes of 27-G and 25-G vitrectomy (no IPVD subgroup analysis) and found 27-G surgeries had improved visual outcomes, reduced intraoperative and postoperative complications, with a slight increase in mean operation time (longer by three minutes) [25]. Despite this, 27-G has struggled to gain widespread adoption, and the 2021 Preferences and Trends Survey of the American Society of Retinal Surgeons revealed 67% surgeons never use 27-G and only 2% routinely use 27-G instruments for vitreoretinal surgery [27]. The findings of our study add supporting evidence to the safety of 27-G instruments in eyes requiring IPVD.

Previous studies have demonstrated an association between higher RB rates and younger age in patients undergoing IPVD, despite finding no association in our results [14]. Rahman et al. investigated a series of 137 patients undergoing IPVD with an age range of 32–94 years (mean 69.9), and revealed each 1 year increase in age was associated with 4.1% decreased odds of a RB (OR 0.959, *p* = 0.028) [14]. Additionally, they found that eyes with an adherent posterior hyaloid, defined as requiring membrane blue with targeted suction, were 3.8 times more likely to experience a RB. Ageing has been associated with partial detachment of PVD, which may lower the suction requirements during IPVD [30]. We may not have found an association in our series given the older age skew and relatively narrow age distribution, limiting the ability to properly examine the effect of age on RB rate. Our conclusions also cannot be generalised to paediatric populations, given these were excluded from our series.

Surgical indication was not associated with RB odds, consistent with existing literature [7]. Chung et al. found no difference in RB rate between eyes undergoing IPVD for ERM or MH surgery (32.1% (9/28) vs. 12.7% (19/105), *p* = 0.0563, respectively) [1]. Rahman et al. also revealed no association between diagnosis and increased RB rate across 137 eyes undergoing vitrectomy [14]. Other studies have shown mixed findings. Nakano et al. demonstrated a higher rate of RB in 84 eyes undergoing MH surgery compared to 38 eyes undergoing ERM (13% vs. 0%), whilst Mura et al. reported ERM may have the highest RB rate across a range of indications (4.2%, 8/189 compared to 1.9%, 2/106 VMTS in the next highest category) [2, 8]. However, these studies do not include an IPVD subgroup analysis.

We found a high proportion (69%) of peripheral RB experienced during IPVD were in the inferior retinal field. Previous studies have reported similar findings. Sandali et al. revealed within eyes requiring IPVD, there was a higher portion of RB that occurred in the inferior retina compared to superior retina, irrespective of gauge size and indication (81.2% vs. 19.0%) [10]. Hikichi et al. also found a significantly higher number of inferior quadrant breaks compared to superior quadrant breaks in eyes requiring IPVD undergoing 23-G surgery (68% vs. 32%, *p* = 0.014) [17]. The proposed mechanism is the physiological direction of PVD. Naturally occurring PVD begins superiorly and extends inferiorly, often resulting in incomplete or partial vitreo-retinal dehiscence in inferior quadrants [17]. Schneider et al. have suggested that as the vitreous undergoes physiological liquefaction and begins detaching, collagen fibrils aggregate into parallel bundles that form thicker adhesions, preventing a complete PVD inferiorly [31]. The higher degree of inferior retinal traction may explain the high inferior RB rate seen in our series of eyes. However, given the exclusion of sclerotomy-related breaks, the greater arc angle inferonasally could introduce a selection bias in the distribution of RBs. Despite this, sclerotomy-related breaks were only experienced in 1.6% of eyes, which is in keeping with previous evidence [2, 32].

A strength of our study is the strict inclusion of eyes which require IPVD, which have previously only been included as a subgroup analysis. However, several limitations should be acknowledged. The retrospective design and relatively small number of cases performed by consultant vitreoretinal surgeons may limit generalizability and overestimate RB rates. Gauge size was not randomized, and its selection is likely contributed to by surgeon preference, case complexity, or perceived RB risk, introducing potential confounding and limits casual inference. Therefore, the associations observed in our analyses should be interpreted as exploratory rather than representative of causal relationships. In addition, several important factors including axial length, refractive status, lattice degeneration, degree of vitreous adherence, and extent of peripheral vitreous shaving were not systematically recorded and therefore could not be included in analyses. These factors are important contributors to retinal break risk and may further confound the observed associations.

## Conclusion

This retrospective study revealed a high rate of RB breaks in eyes undergoing PPV which require IPVD (15%). We found that 25-G compared to 27-G surgery was associated with an increased odd of RBs during IPVD. Other factors including age, lens status, surgical indication and grade of surgeon were not associated with odds of RB. The proportion of breaks located inferiorly was high (69%). Our results add further evidence regarding risk factors for RB during IPVD, although larger, prospective, and ideally randomised studies are needed, and casual inference cannot be drawn from our findings.

## Data Availability

No datasets were generated or analysed during the current study.
